# Nested Quantization Index Modulation for Reversible Watermarking and Its Application to Healthcare Information Management Systems

**DOI:** 10.1155/2012/839161

**Published:** 2011-12-13

**Authors:** Lu-Ting Ko, Jwu-E. Chen, Yaw-Shih Shieh, Hsi-Chin Hsin, Tze-Yun Sung

**Affiliations:** ^1^Department of Electrical Engineering, National Central University, Chungli 320-01, Taiwan; ^2^Department of Electronics Engineering, Chung Hua University, Hsinchu 300-12, Taiwan; ^3^Department of Computer Science and Information Engineering, National United University, Miaoli 360-03, Taiwan

## Abstract

Digital watermarking has attracted lots of researches to healthcare information management systems for access control, patients' data protection, and information retrieval. The well-known quantization index modulation-(QIM-) based watermarking has its limitations as the host image will be destroyed; however, the recovery of medical images is essential to avoid misdiagnosis. In this paper, we propose the nested QIM-based watermarking, which is preferable to the QIM-based watermarking for the medical image applications. As the host image can be exactly reconstructed by the nested QIM-based watermarking. The capacity of the embedded watermark can be increased by taking advantage of the proposed nest structure. The algorithm and mathematical model of the nested QIM-based watermarking including forward and inverse model is presented. Due to algorithms and architectures of forward and inverse nested QIM, the concurrent programs and special processors for the nested QIM-based watermarking are easily implemented.

## 1. Introduction

Digital watermarking is a scheme of embedding data in an image called host image for the purpose of copyright protection, integrity check, and/or access control [1–6]. Some of the requirements of digital watermarking are transparency, robustness, and capacity. Specifically, transparency means that the watermark embedded in the host image is imperceptible to human eyes, robustness means the resistance of the watermark to malicious attacks, and capacity denotes the amount of data that can be hidden in the host image. Digital watermarking has been applied to many applications [7–10].

For medical images, such as radiography, magnetic resonance imaging (MRI), nuclear medicine imaging, photo acoustic imaging, tomography, and ultrasound, the conventional watermarking schemes are not suitable due to the distortion problem, which can lead to misdiagnosis [11–14]. In order to provide the requirements of confidential data protection and intact information retrieval [15–24], watermarking with legal and ethical functionalities is desirable especially for the medial images applications. For diagnostic information retrieving and host image reconstruction, the reversible watermarking can be achieved by using many common used techniques, which are based on the characteristics of nonlinear time series [25–30]. More specifically, confidential data such as patients' diagnosis reports can be taken as watermark data and then embedded in the host image by using digital watermarking with authorized utilization. Thus, digital watermarking can be used to facilitate healthcare information management systems. 

In this paper, we propose a novel scheme called the nested quantization index modulation-(QIM-) based watermarking for the healthcare information management applications. The reminder of the paper proceeds as follows. In Section 2, the conventional QIM-based watermarking is reviewed briefly.  Section 3 describes the nested QIM-based watermarking. Experimental results on medical images are presented in Section 4. Conclusion is given in Section 5.

## 2. Review of Quantization Index Modulation

**Figure 1:**
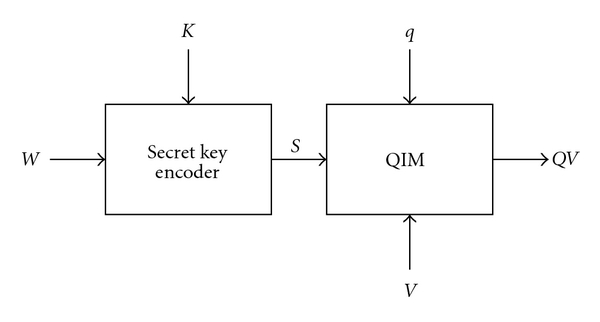
The conventional QIM-based watermarking (*W*: the watermark, *K*: the secret key, *S*: the coded watermark, *q*: the quantization step, *V*: the host image, *QV*: the watermarked image).

**Figure 2:**
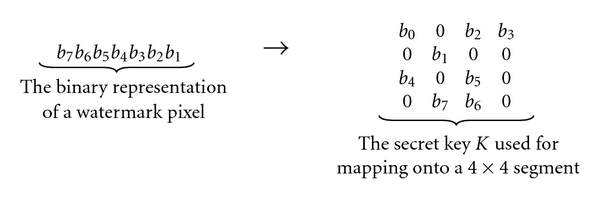
The secret key *K* used for mapping the watermark onto the host image.

Figure 1 depicts the conventional quantization index modulation-(QIM-) based watermarking scheme [31], where *W*, *K*, *S*, *V*, and *QV* denote the watermark, the secret key, the coded watermark, the host image, and the watermarked image, respectively. For the sake of simplicity, let us consider monochromatic images with 256 grey levels, and the size of the watermark is one-fourth of that of the host image. The secret key is used to map the binary representation of the watermark onto the host image, for example, Figure 2 depicts the binary representation of a watermark pixel that is mapped onto a 4 × 4 segment using a given secret key.

**Figure 3:**
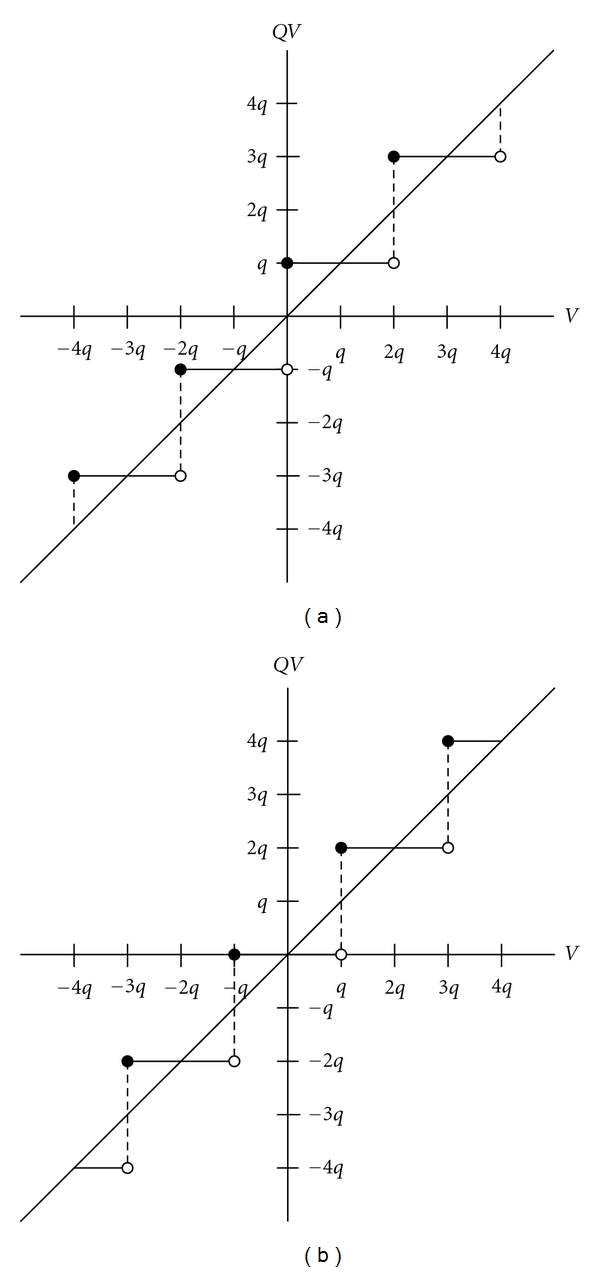
Operations of the QIM scheme for the coded watermark pixels being (a) bit 1 and (b) bit 0, respectively.

Figure 3 shows the operation of the QIM block, in which the grey levels of the host image, *V*, ranging between 2*c* · *q* and (2c + 1) · *q* will be quantized into (2*c* + 1) · *q* if the corresponding pixels of the coded watermark, *S*, are bit 1; otherwise they are quantized into 2*c* · *q* if the corresponding pixels are bit 0. For the grey levels of V that are between (2*c* + 1) · *q* and (2*c* + 2) · *q*, they will be quantized into (2*c* + 1) · *q* or (2*c* + 2) · *q* depending on the corresponding pixels of S being bit 1 or 0, respectively. Note that *q* denotes the quantization step, 0 ≤ *c* < (255/2 · *q*), and *c* is an integer number.

**Figure 4:**
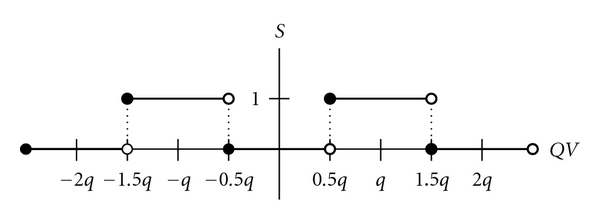
Operations of the inverse QIM scheme for the coded watermark pixels.

**Figure 5:**
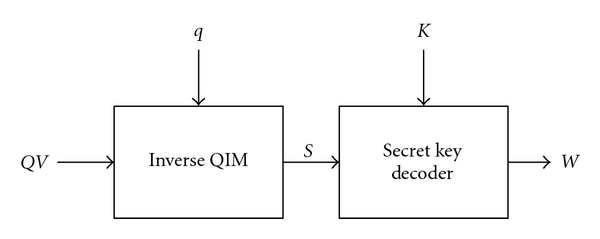
Extraction of the watermark, *W*, from the watermarked image, *QV*, based on the conventional QIM scheme.

It is noted that the watermarked image, *QV*, can be written as

(1) $$QV\left(i,j\right)=\{\begin{array}{cc} \left(2c+1\right)q, & \text{if}\;V\left(i,j\right)\\ & \;\in (\left(2c+0.5\right)q,\left(2c+1.5\right)q],\\ & \;\;\;\;\;\;\;\;\text{S}\left(i,j\right)=1,\\ \left(2c\right)q, & \text{if }V\left(i,j\right)\\ & \;\in (\left(2c-0.5\right)q,\left(2c+0.5\right)q],\\ & \;\;\;\;\;\;\;\;\text{S}\left(i,j\right)=0, \end{array}$$

where (*i*, *j*)  denotes the position index of pixels, and the coded watermark, *S*, can be obtained by

(2) $$S\left(i,j\right)=\{\begin{array}{cc} 1, & \text{if }QV\left(\text{i},\text{j}\right)\in \left(\left(2d+0.5\right)q,\left(2d+1.5\right)q\right],\\ 0, & \text{otherwise,} \end{array}$$

as shown in Figure 4. Together with the secret key, *K*, the watermark, *W*, can be exactly extracted from the watermarked image, *QV*, as shown in Figure 5.

## 3. The Proposed Nested QIM for Reversible Watermarking

**Figure 6:**
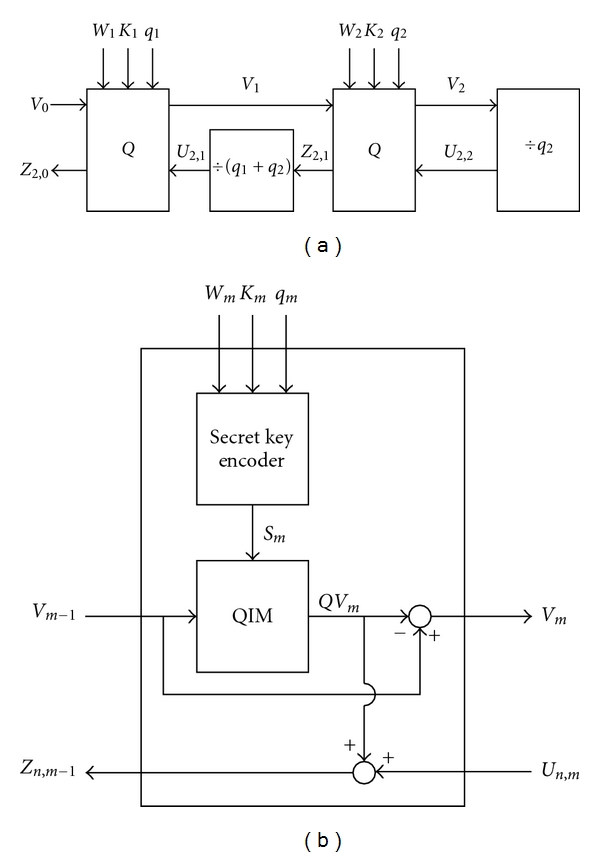
(a) The simplest nested QIM with two *Q* blocks (*V*_0_: the host image, *Z*_2,1_: the intermediate watermarked image, *Z*_2,0_: the final watermarked image, *W*_1_ and *W*_2_: the watermarks, *K*_1_ and *K*_2_: the secret keys, *q*_1_ and *q*_2_: the quantization steps, *V*_1_ and *V*_2_: the quantization errors, *U*_2,1_ and *U*_2,2_: the normalized quantization errors). (b) The *Q* block used in the nested QIM.

One of the fundamental requirements for the medical applications is recovery of the host image. As the conventional QIM-based watermarking is irreversible and the host image can not be exactly reconstructed, we propose a novel algorithm called the nested QIM algorithm for reversible watermarking.  Figure 6(a) depicts the simplest nested QIM consisting of only two QIM operations, each of which is performed in the *Q* block shown in Figure 6(b), where *W*_*m*_, *K*_*m*_, *S*_*m*_, *V*_*m*−1_, *Z*_*n*,*m*−1_, *QV*_*m*_, *V*_*m*_, and *q*_*m*_ are the watermark, the secret key, the coded watermark, the input host image, the output watermarked image, the quantization, the quantization error, and the quantization step at the *m*th stage of a *n*-level nested QIM, respectively. The original host image and the final watermarked image denoted by *V*_0_ and *Z*_2,0_ are taken as the input and the output of the first stage.

As one can see, QIM is a nonlinear function, and we have

(3) $$\begin{array}{c} \begin{array}{c} QV_{1}=\text{QIM}\left(V_{0}\right), \end{array}\\ \begin{array}{c} V_{1}=V_{0}-QV_{1}, \end{array}\\ V_{2}=V_{1}-QV_{2}=V_{1}-\text{QIM}\left(V_{1}\right),\\ \begin{array}{c} Z_{2,1}=QV_{2}+U_{2,2}=QV_{2}+\frac{V_{2}}{q_{2}}. \end{array} \end{array}$$

The final watermarked image, *Z*_2,0_ is thus derived as

(4) $$\begin{array}{cc} Z_{2,0} & {=QV}_{1}+U_{2,1}\\ & {=QV}_{1}+\frac{Z_{2,1}}{q_{1}+q_{2}}\\ & {=QV}_{1}+\frac{QV_{2}+U_{2,2}}{q_{1}+q_{2}}\\ & {=QV}_{1}+\frac{QV_{2}+V_{2}/q_{2}}{q_{1}+q_{2}}. \end{array}$$

**Figure 7:**
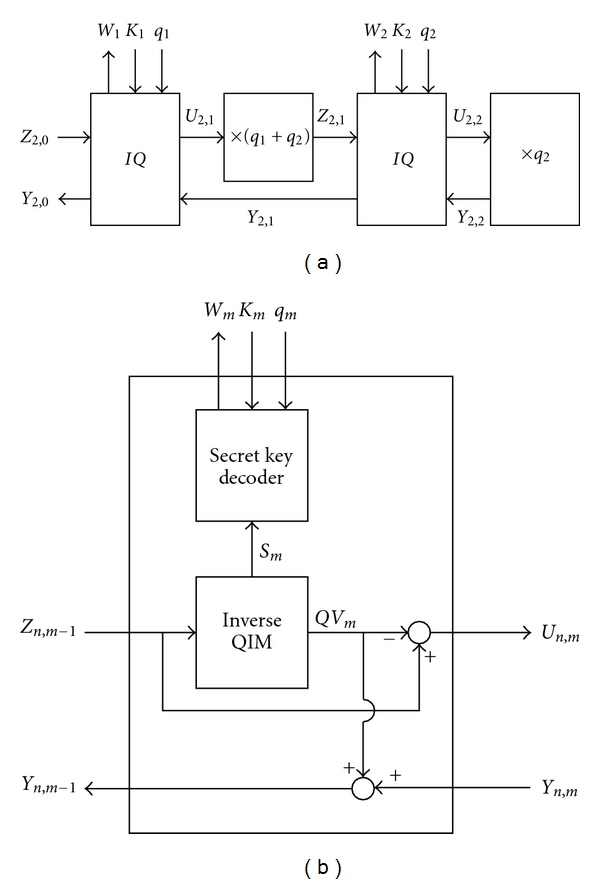
(a) The inverse nested QIM with two inverse *Q*  (*IQ*) blocks. (b) The *IQ* block used in the inverse nested QIM for watermark extraction.

The original host image and the embedded watermark images can be exactly reconstructed from the watermarked image, *Z*_2,0_, by using the inverse nested QIM shown in Figure 7(a), where the inverse *Q*  (*IQ*) block is shown in Figure 7(b).

Based on the data flow of Figures 7(a) and 7(b), we have

(5) $$\begin{array}{c} QV_{1}=\text{IQIM}\left(Z_{2,0}\right),\\ Z_{2,1}=\left(Z_{2,0}-QV_{1}\right)\times \left(q_{1}+q_{2}\right),\\ QV_{2}=\text{IQIM}\left(Z_{2,1}\right),\\ Y_{2,2}=\left(Z_{2,1}-QV_{2}\right)\times q_{2},\\ Y_{2,1}=QV_{2}+Y_{2,2}. \end{array}$$

Together with (3) and (4), the original host image, *V*_0_, is thus obtained by

(6) $$\begin{array}{cc} Y_{2,0} & =QV_{1}+Y_{2,1}\\ & =QV_{1}+QV_{2}+Y_{2,2}\\ & =V_{0}. \end{array}$$

**Figure 8:**
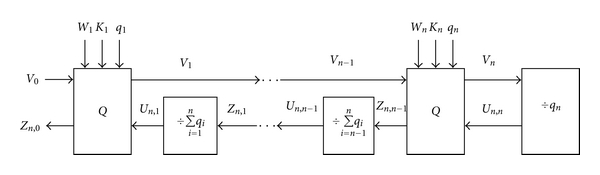
The nested QIM with *n* QIM operations for watermarking.

**Figure 9:**
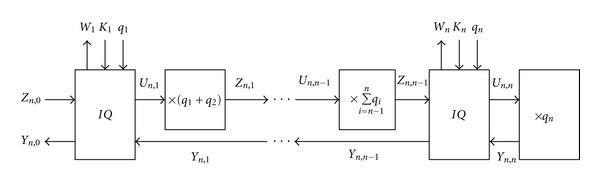
Extraction of the original host and watermark images from the watermarked image based on the nested QIM with *n* inverse QIM operations.

The above-mentioned equations can be generalized as follows:

(7) $$\begin{array}{cc} & QV_{m}=\text{QIM}\left(V_{m-1}\right),\\ & V_{m}=V_{m-1}-QV_{m},\\ & Z_{n,m-1}=QV_{m}+U_{n,m},\\ & Z_{n,0}=QV_{1}+\frac{V_{n}-QV_{n}}{\prod_{h=1}^{n}\left(\sum_{k=h}^{n}q_{k}\right)}+\overset{n}{\underset{j=2}{\sum }}\frac{QV_{j}}{\prod_{h=1}^{j-1}\left(\sum_{k=h}^{n}q_{k}\right)}, \end{array}$$

(8) $$\begin{array}{cc} & QV_{m}=\text{IQIM}\left(Z_{n,m-1}\right),\\ & U_{n,m}=Z_{n,m-1}-QV_{m},\\ & Y_{n,m-1}=Y_{n,m}+QV_{m},\\ & \begin{array}{cc} Y_{n,0} & =QV_{1}+Y_{n,1}\\ & =QV_{1}+QV_{2}+\cdots +QV_{m}+Y_{n,n}\\ & =\left(\overset{n}{\underset{k=1}{\sum }}QV_{k}\right)+Y_{n,n}\\ & =V_{0}. \end{array} \end{array}$$

where (7) are used for the nested QIM, and (8) for the inverse nested QIM. The corresponding block diagrams for embedding and extracting watermarks with the host image are shown in Figures 8 and 9, respectively.

## 4. Experimental Results on Medical Images

**Figure 10:**
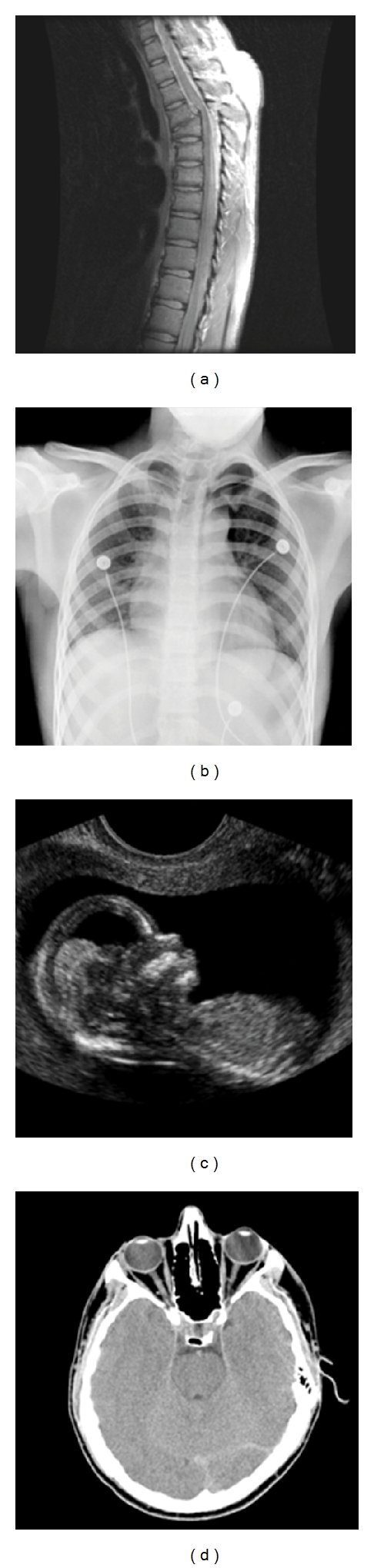
The 256 × 256 host images with 256 grey levels; (a) spine (MRI), (b) chest (X-ray), (c) fetus (ultrasonic), and (d) head (CT).

**Figure 11:**
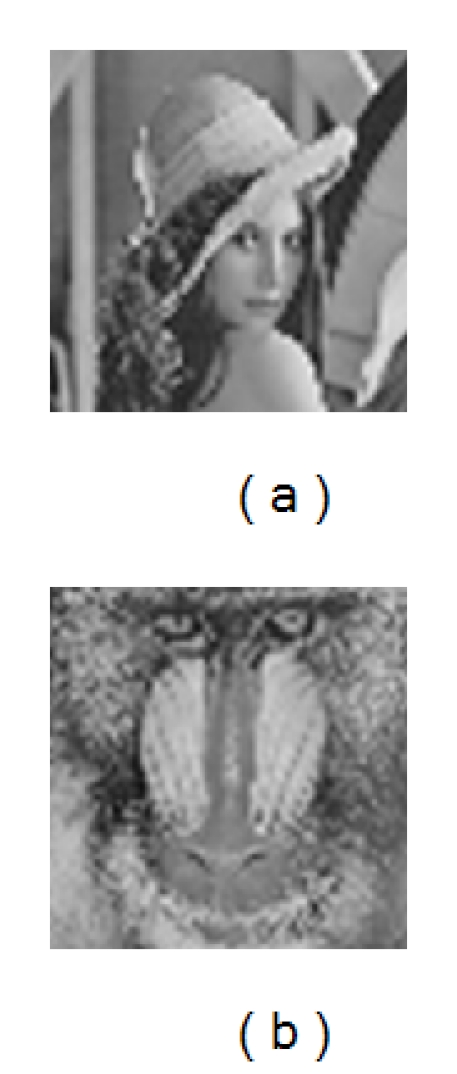
The 64 × 64 (a) Lena image and (b) Baboon image with 256 grey levels used as watermarks.

The nested QIM-based watermarking algorithm has been evaluated on various medical images.  Figure 10 shows the test 256 × 256 images with 256 grey levels, namely, spine, chest, fetus and head obtained by magnetic resonance image (MRI), X-ray, ultrasound, and computed tomography (CT), respectively, which are used as host images.  Figure 11 shows the 64 × 64 Lena image and Baboon image with 256 grey levels, which are used as watermarks.

**Figure 12:**
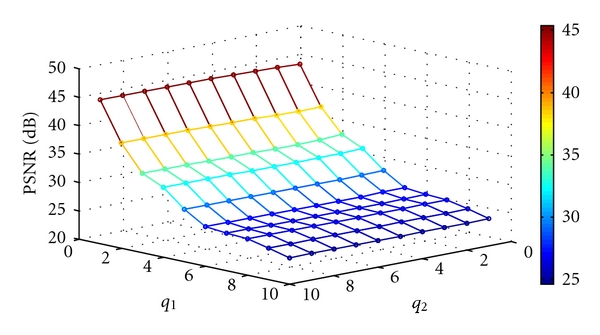
The PSNR of the watermarked image of spine (MRI) at various quantization steps *q*_1_ and *q*_2_.

**Figure 13:**
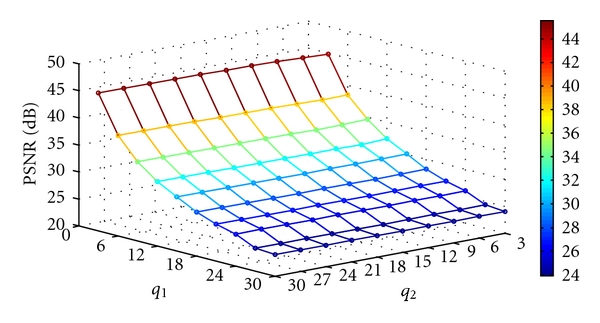
The PSNR of the watermarked image of chest (X-ray) at various quantization steps *q*_1_ and *q*_2_.

**Figure 14:**
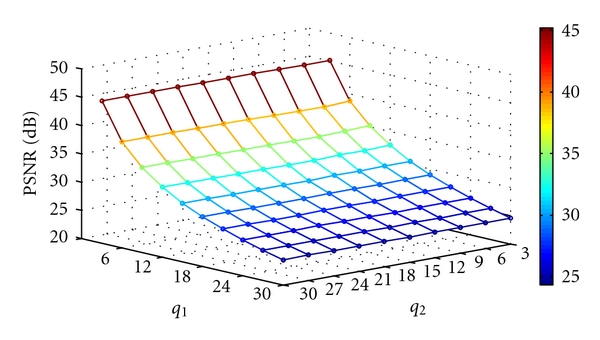
The PSNR of the watermarked image of fetus (ultrasonic) at various quantization steps *q*_1_ and *q*_2_.

**Figure 15:**
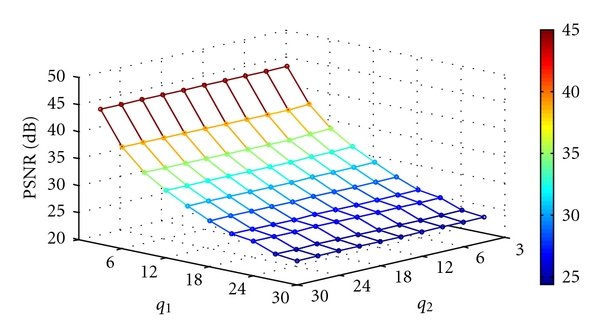
The PSNR of the watermarked image of head (CT) at various quantization steps *q*_1_ and *q*_2_.

**Figure 16:**
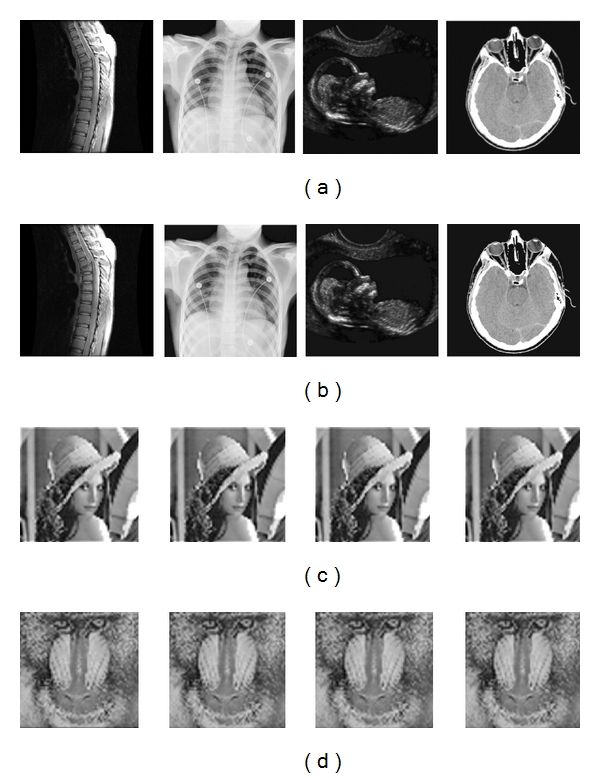
The watermarked images (a), the reconstructed images (b), and the extracted watermarks ((c) and (d)) with the quantization steps *q*_1_ = 30 and *q*_2_ = 30.

The peak signal to noise ratio (PSNR) is used to evaluate the image quality [18, 31], which is defined as

(9) $$\text{PSNR}=20\log\left(\frac{255}{\sqrt{\text{MSE}}}\right),$$

where MSE denotes the mean square error. Figures 12, 13, 14, and 15 show the PSNR of the watermarked images of spine (MRI), chest (X-ray), fetus (ultrasonic), and head (CT) at various quantization steps, *q*_1_ and *q*_2_. Figure 16 shows the watermarked images (first row), the reconstructed images, (second row) and the extracted watermarks (third and fourth rows) with *q*_1_ = 30 and *q*_2_ = 30. It is noted that the watermarked images even with large quantization steps are almost indistinguishable from the exactly reconstructed host images.

## 5. Conclusion

In this paper, a novel algorithm called the nested QIM has been proposed for medical image watermarking. The capacity of the embedded watermark can be increased by taking advantage of the proposed nest structure. As the host image can be exactly reconstructed, it is suitable especially for the medical image applications. In addition, the healthcare information such as patients' data, digital signatures, and identification codes can be well embedded in medical images. Thus, the nested QIM-based medical image watermarking is preferable to facilitate data management in healthcare information management systems.
